# Brain Dysfunction in LAMA2-Related Congenital Muscular Dystrophy: Lessons From Human Case Reports and Mouse Models

**DOI:** 10.3389/fnmol.2020.00118

**Published:** 2020-07-23

**Authors:** Andrea J. Arreguin, Holly Colognato

**Affiliations:** ^1^Department of Pharmacological Sciences, Stony Brook University, Stony Brook, NY, United States; ^2^Medical Scientist Training Program (MSTP), Stony Brook University, Stony Brook, NY, United States

**Keywords:** LAMA2, MDC1A, brain development, congenital muscular dystrophy, dystroglycanopathies, laminin

## Abstract

Laminin α2 gene (LAMA2)-related Congenital Muscular Dystrophy (CMD) was distinguished by a defining central nervous system (CNS) abnormality—aberrant white matter signals by MRI—when first described in the 1990s. In the past 25 years, researchers and clinicians have expanded our knowledge of brain involvement in LAMA2-related CMD, also known as Congenital Muscular Dystrophy Type 1A (MDC1A). Neurological changes in MDC1A can be structural, including lissencephaly and agyria, as well as functional, including epilepsy and intellectual disability. Mouse models of MDC1A include both spontaneous and targeted LAMA2 mutations and range from a partial loss of LAMA2 function (e.g., *dy^2J^/dy^2J^*), to a complete loss of LAMA2 expression (*dy*^3K^/*dy*^3K^). Diverse cellular and molecular changes have been reported in the brains of MDC1A mouse models, including blood-brain barrier dysfunction, altered neuro- and gliogenesis, changes in synaptic plasticity, and decreased myelination, providing mechanistic insight into potential neurological dysfunction in MDC1A. In this review article, we discuss selected studies that illustrate the potential scope and complexity of disturbances in brain development in MDC1A, and as well as highlight mechanistic insights that are emerging from mouse models.

## Introduction

### Laminin Structure and Function

Laminins are developmentally regulated extracellular matrix (ECM) molecules comprised of α, β, and γ chains. Laminins play critical roles in the establishment and overall organization of basement membranes (BMs) as well as in controlling signaling pathways through interactions with cell surface receptors. All laminins exist as heterotrimers comprised of one α, one β, and one γ chain, assembled via a triple coiled-coil, and are named based on their composition, e.g., Laminin-111 contains the α1, β1, and γ1 chains. Five genes encode the α1–α5 chains (LAMA1 to LAMA5), four genes encode the β1–β4 chains (LAMB1-LAMB4) and three genes encode the γ1–γ3 chains (LAMC1–3; Yurchenco et al., 2018). In most laminin trimers, the three N-terminal regions are “free,” i.e., they extend beyond the coiled-coil region, producing a cross-shaped molecule with N-terminal short arms. Laminins with a full complement of three N-terminal short arms can form an inter-laminin meshwork, or polymer, while laminins without short arms (e.g., laminins with α3A or α4 subunits) do not polymerize (Durbeej, 2010; Yurchenco et al., 2018). Importantly, the C-terminal α-subunit of all laminins, which extends beyond the coiled-coil region, contains binding sites for the majority of laminin-binding receptors. These differing structural properties, in concert with varying abilities to bind cell surface receptors and other ECM proteins such as nidogen and perlecan, are thought to underlie the ability of different laminins to convey differing chemical and mechanical signals to cells.

Receptors for laminins include integrins, most prominently integrins α6β1 and α7β1. Integrins are transmembrane glycoprotein receptors that mediate cellular and ECM interactions as well as activate signal transduction pathways (Belkin and Stepp, 2000). α7β1 is a critical receptor of laminin in developing and adult skeletal muscle and loss of α7β1 leads to mild muscular dystrophy (Saher and Hildt, 1999). The interaction between LAMA2 loss and α7β1 is discussed in depth in another review of this special issue (Barraza-Flores et al., 2020). Laminin-binding integrins mediate signaling responsible for developmental processes in the central nervous system (CNS) such as axon outgrowth (Tomaselli et al., 1988) and cell migration (Desban and Duband, 1997). Other non-integrin binding partners include dystroglycan (Brancaccio et al., 1995; Colognato and Yurchenco, 2000) and heparan sulfate proteoglycans (Brown et al., 1994). Dystroglycan, a transmembrane receptor of the Dystrophin-Glycoprotein Complex (DGC), is comprised of α- and β-subunits that remain tightly associated (Moore and Winder, 2012). α-Dystroglycan, which is entirely extracellular, undergoes extensive glycosylation that is necessary for α-dystroglycan to directly interact with laminin-like G (LG) domain-containing ligands, which include laminins, agrin, and perlecan, while β-dystroglycan is transmembrane and interacts with multiple binding partners within the cell (Moore and Winder, 2012). For instance, laminin-binding to α-dystroglycan in skeletal muscle anchors the ECM to the actin cytoskeleton via the interaction of β-dystroglycan with the actin-binding protein, dystrophin (Yurchenco et al., 2018). Disturbances in α-dystroglycan glycosylation, which impair binding to LG domain-containing extracellular ligands including laminin, underlie a collection of genetic disorders known as *α*-dystroglycanopathies (Jimenez-Mallebrera et al., 2005).

LAMA2 encodes for the α2 chain and mutations in the LAMA2 gene can disrupt either the expression or the binding capacity of α2-containing laminins such as laminin-211 (Lm-211). This loss-of-function is best understood in skeletal muscle, where Lm-211, via interactions with dystroglycan and α7β1 integrin receptors, is critical for proper BM assembly and function, which in turn is needed for the stability of the muscle sarcolemma (Yurchenco et al., 2018). While muscle pathology is described in detail in other reviews of this issue (Accorsi et al., 2020; Barraza-Flores et al., 2020; Gawlik and Durbeej, 2020), in general these abnormalities include apoptosis, fibrosis, inflammation. However laminin-α2 deficiency in muscle also leads to an upregulation of Lm-411 (α4, β1, γ1; Patton et al., 1997), and Lm-511 (α5, β1, γ1), which normally disappear postnatally, remaining only at the neuromuscular junction (NMJ) in healthy adult muscle (Patton et al., 1997; Kölbel et al., 2019). Several lines of evidence suggest that these laminins may not functionally compensate for Lm-211. For instance, while Lm-511 can polymerize, Lm-411 cannot (Durbeej, 2010; Di Russo et al., 2017; Yurchenco et al., 2018); and, while Lm-411 and Lm-511 both bind to α3β1 and α6β1 integrins (Fujiwara et al., 2001), there is no direct evidence to suggest that Lm-411 and Lm-511 bind to α-dystroglycan strongly enough to provide a fully functional mechanical “ECM-to-cytoskeletal bridge” for contracting muscle (Yu and Talts, 2003; Reinhard et al., 2017). One study, using various combinations of knockout mouse models, found that only a double mutant (Lama4^−/−^; Lama5^M/M^), but not single mutants, resulted in less clustering of dystroglycan at the NMJ (Nishimune et al., 2008). In a different study that more directly assessed α4 and α5 binding to α-dystroglycan, both laminins were able to bind but with low affinity and only in the presence of linker proteins, including mini-agrin (Reinhard et al., 2017). Lastly, in a study that compared muscle integrity in a dystroglycan-deficient mouse (Dag1^−/−^) vs. an α7β1 deficient mouse (Itga7^−/−^), the BM only detached in the Dag1^−/−^ mouse (Han et al., 2009), indicating the importance of the laminin-dystroglycan connection in the sarcolemmal BM. These experiments and others suggest that laminins that contain α4 and α5 chains are not able to fully compensate for the loss of laminin-α2 expression, especially regarding its binding to α-dystroglycan. However, a full understanding of the cell and tissue phenotypes that occur in the absence of normal LAMA2 expression remains challenging, as phenotypes may arise from a complex mixture of loss- and gain-of-functions.

The ability of receptors to interact with α2-containing laminins is also critical in the CNS, where the loss of laminin-α2 results in brain abnormalities that include neuronal migration defects that can result in lissencephaly. Interestingly, in severe cases of α-dystroglycanopathies, there are gross cortical lamination abnormalities, resulting in profound neurological deficits; these more extreme deficits are more rarely observed in congenital muscular dystrophy type 1A (MDC1A). These and other differences described ahead suggest that MDC1A and dystroglycanopathies may have some shared, but some distinct, cellular mechanisms underlying their respective CNS pathologies. As in muscle, LAMA2-related phenotypes in the brain are likely to be a complex mixture of functional loss coupled with an altered ECM landscape.

### Congenital Muscular Dystrophy and LAMA2 Mutations

**Figure 1:**
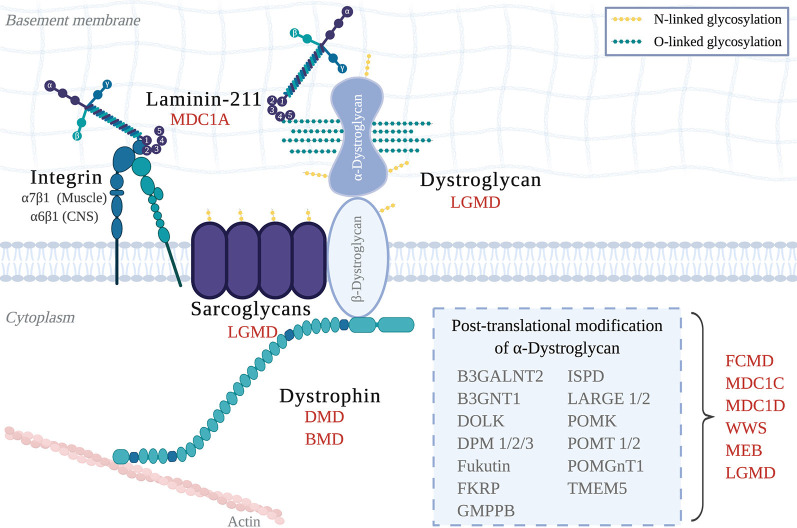
The Dystrophin-Glycoprotein Complex (DGC) in skeletal muscle and its related muscular dystrophies. Abbreviations: MDC1A, Congenital Muscular Dystrophy Type 1A; LGMD, Limb-Girdle Muscular Dystrophy; DMD, Duchenne Muscular Dystrophy; BMD, Becker Muscular Dystrophy; FCMD, Fukuyama Congenital Muscular Dystrophy; MDC1C, Congenital Muscular Dystrophy Type 1C; MDC1D, Congenital Muscular Dystrophy Type 1D; WWS, Walker–Warburg Syndrome; MEB, Muscle–eye–brain disease.

Congenital muscular dystrophies (CMD) are a collection of heterogeneous genetic disorders that largely result from mutations in genes required for the DGC. Subtypes of CMD include MDC1A, Walker–Warburg Syndrome (WWS), Muscle–Eye–Brain disease (MEB), and additional dystroglycanopathies (Johnson et al., 2018; Figure 1). CMD patients present with symptoms that include hypotonia, muscle weakness, and elevated serum creatine kinase (CK), of which onset occurs at birth or early infancy (Mackay et al., 1998). If these symptoms are present at birth, they are collectively called “floppy infant” syndrome, which is highly suggestive of neuromuscular disease (e.g., spinal muscular atrophy, CMD), and require further testing. Pathological features of all CMDs include extensive muscle wasting, necrosis, and fibrosis, and most, if not all, CMDs can also present with CNS involvement, which includes white matter abnormalities, structural brain abnormalities (e.g., cortical dysplasias; see Figure 3), and ocular involvement (Jimenez-Mallebrera et al., 2005).

**Figure 2:**
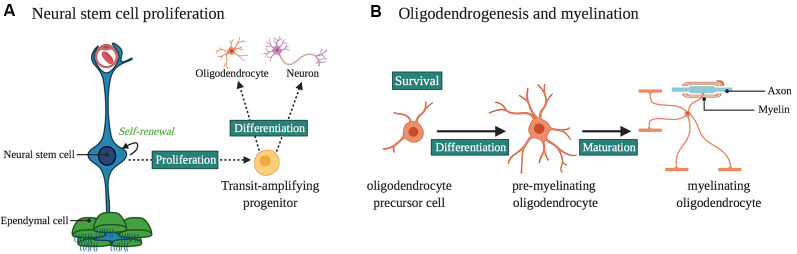
Laminin α2 in the ventricular-subventricular zone (VZ-SVZ) neural stem cell (NSC) niche. **(A)** Laminin α2 influences NSC proliferation and differentiation. It influences neurogenesis and gliogenesis in the VZ-SVZ, as well as the proliferation and survival of progenitors for midbrain dopaminergic neurons. **(B)** Oligodendrogenesis and myelination are influenced by LAMA2 expression. In particular, LAMA2 expression influences the survival and differentiation of oligodendrocyte precursor cells (OPCs) as well as the maturation of oligodendrocytes.

**Figure 3:**
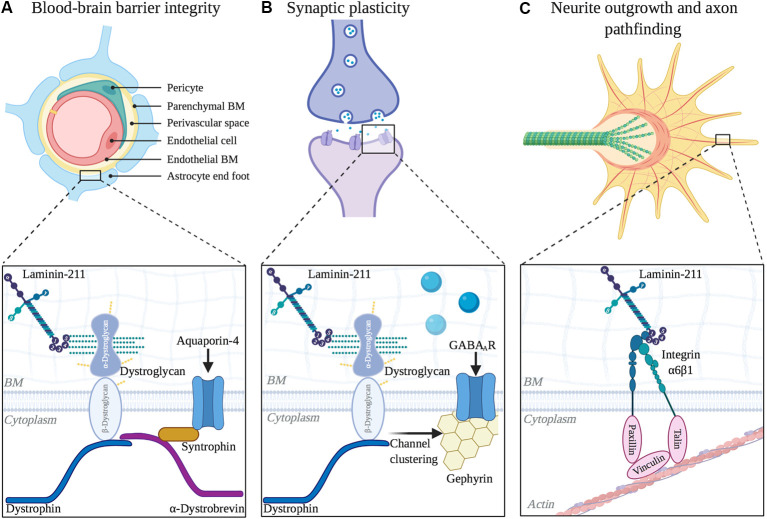
Functional roles of laminin α2 in other areas of the central nervous system (CNS). **(A)** Blood-brain barrier integrity is regulated by LAMA2 expression. Inset depicts laminin interaction with dystroglycan, which helps to cluster aquaporin-4 channels at barrier astrocytic endfeet. **(B)** Laminin α2 influences long-term synaptic plasticity. Inset depicts laminin interaction with dystroglycan, which helps to mediate the clustering of GABA_A_ receptors at inhibitory synapses. **(C)** Neurite outgrowth and axon pathfinding are influenced by α2-containing laminins. Inset depicts laminin interaction with integrin α6β1 in neuronal growth cones, which helps to couple extracellular matrix (ECM) and the actin cytoskeleton as growth cones extend.

While over 20 genes have been implicated in CMDs, and CMDs can share clinical characteristics, this review article will focus on features of MDC1A from human and mouse model studies, as well as compare MDC1A to CMDs arising from mutations in Lm-211 binding partners. MDC1A, while still classified as a rare disease, is the most common form of CMD in many regions (Allamand et al., 1997; Sframeli et al., 2017; Ge et al., 2019). MDC1A is considered the classical CMD as nearly half of all CMD patients lack or express reduced levels of laminin-α2 in muscle BM (Cohn et al., 1998). Patients with MDC1A (previously referred to as merosin-deficient CMD as merosin was the original name for α2-containing laminins) also represent a heterogeneous group. For instance, two large studies have revealed a correlation between the severity of MDC1A and the degree of laminin-α2 expression (Allamand et al., 1997; Geranmayeh et al., 2010). In one study of 51 MDC1A cases, the *complete absence* of laminin-α2 correlated with worse clinical outcomes and earlier presentation of the disease when compared to a *partial loss* of laminin-α2 (Geranmayeh et al., 2010). This study highlights the importance of determining the status of laminin-α2 levels in patients with MDC1A, as laminin-α2 levels appear to correlate with the severity of the muscle phenotype. In the following sections, we will highlight both typical and atypical CNS findings in patients with MDC1A.

## Central Nervous System Involvement in MDC1A

### Diffuse White Matter Changes by MRI

Although only a subset of MDC1A patients have profound neurological deficits, white matter changes (i.e., hypointensity on T1 weighted imaging) are routinely found in all MDC1A patients such that the presence of these findings increases the clinical suspicion of MDC1A (Allamand et al., 1997; Geranmayeh et al., 2010; Bönnemann et al., 2014). In a study of 20 patients with the clinical diagnosis of CMD, 13 patients had complete absence or deficiency of laminin-α2, with all 13 having white matter abnormalities upon MRI (Herrmann et al., 1996). Although genetic testing was not performed in these patients, the clinical suspicion for MDC1A is high because of the white matter abnormalities seen in these 13 patients. In another MDC1A study (referred to as classic CMD in the study) in which 9 out of 15 patients were laminin-α2 deficient, disease onset occurred in the first year of life, and all nine had substantial white matter changes that resembled periventricular white matter involvement seen in leukodystrophy (Lamer et al., 1998). Also, seven out of nine laminin-α2 deficient patients had pons hypoplasia and one had evidence of cortical atrophy, likely a result of failed cortical growth (Lamer et al., 1998). In a larger MRI study of 21 MDC1A patients, all but three had white matter changes (Geranmayeh et al., 2010). However, since the MRIs in these three patients were done neonatally, and MRIs of MDC1A patients can appear normal in the first 6 months of age but go on to become abnormal, it remains unclear if these patients were in fact truly lacking white matter changes. The same study reported that while 18 out of 21 patients with MDC1A had typical white matter changes, 13 out of 21 had additional brain abnormalities that included ventricular dilation, cortical dysplasia and atypical white matter changes (Geranmayeh et al., 2010). These numbers indicate that changes beyond the typical white matter changes in MDC1A are not rare, but instead are fairly common.

While it is largely known that MDC1A is accompanied by diffuse brain white matter changes, the underlying pathogenesis remains unclear. One hypothesis is that these white matter abnormalities are due to increased water content due to an impaired selective filtration caused by laminin-α2 deficiency (Villanova et al., 1996, 1997; Menezes et al., 2014). A second hypothesis is that these white matter changes indicate structural changes in the white matter tracts themselves, since interactions between laminin-α2 containing laminins and integrins on developing oligodendrocytes (myelinating cells of the CNS) enhance the development of myelin membrane (Buttery and ffrench-Constant, 1999; Chun et al., 2003; Barros et al., 2009; Relucio et al., 2009, 2012). Ahead we will describe studies that support both hypotheses.

### Reduced Fractional Anisotropy on Diffusion Tensor Imaging

Fractional anisotropy (FA) is a metric of diffusion that is sensitive to aligned obstacles such as cell membranes and myelin (Mori and Zhang, 2006). In the brain, a white matter region with a low FA reflects a lack of coherent fiber organization, which is often due to myelin deficits but can also be due to other structural problems such as an abnormal axon packing and organization. However, FA is used as a readout for white matter maturation, as the typical time course in both mice (Mori and Zhang, 2006) and humans (Keunen et al., 2018) demonstrates that during the perinatal period FA increases in white matter tracts as resident axons become increasingly myelinated. In one case report of a 6-year-old MDC1A patient with gross developmental delay, along with typical diffuse white matter hyperintensities on T2 weighted MRI, diffusion tensor imaging (DTI) revealed a reduced FA (Ip et al., 2012).

In a second case report of a 6-month-old MDC1A patient with hypotonia and delayed motor development, MRI at the age of 5 years old revealed the typical diffuse white matter abnormalities (Sijens et al., 2007). By 10 years old, the patient developed epilepsy, cognitive regression and behavioral problems which eventually required hospitalization at 15 years old, where DTI revealed reduced FA in both the gray and white matter (Sijens et al., 2007). Unfortunately, due to a lack of DTI studies in MDC1A patients, it is currently unclear whether a low FA metric, a hallmark of poor white matter maturation, is characteristic of the MDC1A or just found in these isolated cases.

### Prevalence of Focal Cortical Dysplasia and Epilepsy in MDC1A

While not as universal as white matter abnormalities, epilepsy and cortical dysplasias (i.e., polymicrogyria and lissencephaly) also occur in patients with MDC1A (Herrmann et al., 1996; Brett et al., 1998; Vigliano et al., 2009; Geranmayeh et al., 2010; Gavassini et al., 2011; Marques et al., 2014). In one example, a case report describes a child with floppy infant symptoms at birth with absent laminin-α2 muscle staining and an abnormal brain MRI with diffuse white matter abnormalities and cortical dysplasia in the occipital lobe (Brett et al., 1998). Another report describes an infant who was diagnosed with MDC1A at 5 months, without any notable CNS involvement, but progressively developed CNS symptoms that began with accidental falls at 4 years old, and eventually was diagnosed with epilepsy at 6 years old (Vigliano et al., 2009). Focal cortical dysplasia is a developmental neuroanatomical malformation that frequently leads to refractory epilepsy, and an MRI study of this patient after the first seizure revealed occipital polymicrogyria. Three MDC1A patients with focal cortical dysplasias and refractory occipital epilepsies that began in early childhood were described in a separate report, with two patients having normal intellect and one patient having moderate intellectual disability (Pini et al., 1996).

Another report described five patients with LAMA2 mutations, of which two patients had epilepsy and three patients had more subtle neurological symptoms (Gavassini et al., 2011). One patient had refractory epilepsy, and interestingly only had “mild muscle weakness” (Gavassini et al., 2011). This case suggests that the severity of the CNS and muscular dystrophy phenotypes do not necessarily correlate. One possible explanation for this lack of correlation is that, depending on the tissue type, a laminin-α2 deficiency could be differentially compensated for, either by other laminins or by other mechanisms entirely.

Another example of the discrepancy between the CNS and muscle symptoms in MDC1A is a case report of a girl with a partial laminin-α2 deficiency with delayed motor development, moderate intellectual disability and severe epileptic seizures (Deodato et al., 2002). At 6 years of age, she underwent neurological evaluation and a CT scan revealed white matter changes typical of MDC1A (diffuse hypointensity on T1 weighted imaging). Examination also revealed peripheral neuropathy and bilateral reduction in amplitude and increased latency of visual evoked potentials (Deodato et al., 2002). At 19 years of age, her MRI revealed similar diffuse white matter changes and she still had severe epilepsy, which had become refractory (Deodato et al., 2002). Despite these relatively severe CNS symptoms for MDC1A, she had normal strength in all proximal muscle, and no muscle wasting or contractures, with only slight difficulty climbing stairs (Deodato et al., 2002).

In another case of an MDC1A patient with extensive CNS involvement, macrocephaly was detected before 1 year of age and refractory epilepsy developed by 6 years of age (Marques et al., 2014). MRI revealed agyria in the occipital cortex, along with white matter abnormalities and widening of frontal gyri, which is consistent with structural abnormalities observed in other cases of laminin-α2 deficiency (Marques et al., 2014). Remarkably, although on muscle biopsy there was irregular laminin-α2 immunofluorescence staining, the patient did not present with any neuromuscular or cardiac complaints, even upon follow-up at 21 years of age (Marques et al., 2014). Sequencing of the LAMA2 gene revealed two heterozygous missense mutations in the N-terminus, one in exon 5 (LN domain) and one in exon 18 (LEb domain). This is an additional case demonstrating that the severity of the neurological and the muscular symptoms do not have to correlate. It is important to note that in all of these MDC1A cases with a discrepancy in the severity of muscle vs. neurological symptoms, patients had reduced laminin-α2 expression (immunofluorescence and protein blots), but not a complete loss. Intriguingly these cases suggest that certain LAMA2 mutations can present largely as a neurological disorder, thus expanding the clinical spectrum of MDC1A.

Epilepsy frequently affects patients with MDC1A and a more recent study found that 9 out of 25 MDC1A patients had epilepsy with a mean age onset of first seizure at 8 years of age (Natera-de Benito et al., 2020). MRI studies of 19 patients revealed that all had white matter abnormalities and that polymicrogyria was found in all epilepsy patients (Natera-de Benito et al., 2020). Although MDC1A-related white matter changes are typically seen in patients of at least 6 months of age, this study revealed that four patients under 6 months of age already had white matter changes, including two in the perinatal period (Natera-de Benito et al., 2020).

### Neuronal Migration Defects

While moderate or subtle changes in CNS anatomy are common in MDC1A, gross structural abnormalities are less frequent, but do occur. In a brain imaging study of 14 patients with MDC1A, all had typical white matter changes consistent with laminin-α2 deficiency, while within the subset of patients that demonstrated gross structural brain abnormalities, on muscle biopsy all had complete loss of laminin-α2 immunofluorescence protein labeling (Philpot et al., 1999). Structural changes included hypoplasia of the cerebellar vermis (5 out of the 10 with complete loss of laminin-α2), cerebellar hemisphere (4 out of the 10) and the pons (3 out of the 10; Philpot et al., 1999). One patient had involvement in U fibers, which are connections between adjacent gyri (Philpot et al., 1999). Another patient, who had epilepsy and moderate intellectual disability, also demonstrated occipital agyria, also consistent with a neuronal migration defect (Philpot et al., 1999).

In another study, an MRI of a 6-week old patient revealed diffuse white matter hypointensity and left occipital lobe pachygyria and agyria (Mackay et al., 1998). An electroencephalogram (EEG) performed at 7 months was abnormal, with isolated sharp components and occipital lobe spike upon photic stimulation (Mackay et al., 1998). However, by 17 months of age, this patient did not have any evidence of seizures (Mackay et al., 1998). A second patient of 2 years of age also exhibited bilateral occipital lobe pachygyria and polymicrogyria, as well as gray matter heterotopia, which is a neuronal migration defect that results in the abnormal localization of neurons. Similar to the first patient, despite these structural defects and an abnormal EEG, this patient was seizure-free (Mackay et al., 1998). Lastly, another report (Sunada et al., 1995) described two cases of MDC1A with severe muscular dystrophy and brain structural abnormalities. The first patient had abnormal gyral formations of the occipital lobe, suggestive of polymicrogyria, and white matter abnormalities within the corpus callosum and centrum semiovale, a large white matter area beneath the cerebral cortex (Sunada et al., 1995). In the occipital lobe, the patient also had areas that resembled hamartomas, which are often due to neuronal migration or overgrowth defects (Sunada et al., 1995). The second patient had polymicrogyria of the posterior temporal, parietal, and occipital lobes and white matter abnormalities of the supratentorial white matter (Sunada et al., 1995). Overall these and other studies reveal that defects in neuronal migration are not uncommon in MDC1A, demonstrating the importance of laminin-α2 expression to brain development. Yet there remains an incomplete understanding of spatial and temporal LAMA2 expression in the developing brain. In the next section, we will review what is known regarding laminin-α2 expression in the CNS, highlighting areas with relevance to MDC1A CNS phenotypes.

## LAMA2 Expression in the Brain and Spinal Cord

During CNS development, laminins are broadly expressed, influencing neurite outgrowth (Morissette and Carbonetto, 1995; Powell et al., 1998), synaptogenesis (Tian et al., 1997) and myelination (Buttery and ffrench-Constant, 1999; Chun et al., 2003; Relucio et al., 2009, 2012; De La Fuente et al., 2017). In the adult brain, the distribution and the expression of different laminins is restricted, for instance to regions such as the basal lamina of cerebral blood vessels and the retina (Morissette and Carbonetto, 1995; Toti et al., 1997; Villanova et al., 1997). Table 1 summarizes the expression of laminin-α2 in the CNS. All of the studies included in Table 1 used antibodies specific for the laminin-α2 chain. This is not likely to be an exhaustive list of the LAMA2 expression given that many studies (not included in Table 1) use antibodies that detect other laminin subunits that may be present in α2-laminin containing trimers. For example, many studies use antibodies that detect the laminin γ1 subunit, which is found in Lm-211 but also in a large subset of laminins, thus making it impossible to know which laminin trimer is present without further analysis. In the following section, we will describe the roles of laminins during brain development and discuss their lesser-known roles in the postnatal and adult brain. In some cases, laminin-α2 association has been clearly described, and in some cases, laminins more broadly as a family have been implicated.

**Table 1:** Sites of laminin-α2 expression in the developing and adult central nervous system.

Location	Embryonic	Postnatal*	Adult**
Astrocytes at the blood-brain barrier	Not available	**Immunofluorescence** *Human*: - α2 chain mouse mAb, 80 kDa specific; α2 chain rat mAb, 300 kDa specific (Villanova et al., 1997). *Mouse*: - α2 chain rat mAb, N-terminus specific (Menezes et al., 2014) **Protein, western blotting** *Mouse*: - α2 chain rat mAb, N-terminus specific (Menezes et al., 2014)	**Immunofluorescence** *Human*: - α2 mouse mAb, 80 kDa specific, and α2 rat mAb, 300 kDa specific (Villanova et al., 1997). *Mouse*: - α2 chain rat mAb, N-terminus specific (Menezes et al., 2014) - α2 chain mouse mAb, 300 kDa specific (Hannocks et al., 2018) - α2 chain mouse mAb, 300 kDa specific (Sixt et al., 2001) - α2 chain rat mAb, N-terminus specific (Yao et al., 2014)
Cortical plate	**Immunofluorescence** *Mouse*: - α2 chain (Campos et al., 2004) - α2 chain rat, N-terminus specific (Lathia et al., 2007)	Not available	Not available
Choroid plexus	**RNA, northern hybridization** *Human*: - α2 chain, multiple nucleotide residues screened using previously reported cDNA (Vuolteenaho et al., 1994)	Not available	Not available
Cerebellum	**RNA, northern hybridization** *Human*: - α2 chain, multiple nucleotide residues screened using previously reported cDNA (Vuolteenaho et al., 1994)	**Immunofluorescence** *Mouse*: - α2 chain mouse mAb, N-terminus (Powell et al., 1998) **Protein, western blotting** *Mouse*: - α2 chain mouse mAb, N-terminus (Powell et al., 1998)	Not available
Hippocampus	**Protein, western blotting** *Rat*: - α2 chain rabbit polyclonal Ab, C-terminus domain G4/5 specific (Tian et al., 1997)	**Immunofluorescence** *Rat*: - α2 chain rabbit polyclonal Ab, C-terminus domain G4/5 specific (Tian et al., 1997)	**Immunofluorescence** *Monkey*: - α2 chain rabbit polyclonal Ab, C-terminus domain G4/5 specific (Hagg et al., 1997) *Rabbit*: - α2 chain rabbit polyclonal Ab, C-terminus domain G4/5 specific (Hagg et al., 1997) *Rat*: - α2 chain rabbit polyclonal Ab, C-terminus domain G4/5 specific (Tian et al., 1997) - α2 chain rabbit polyclonal Ab, C-terminus domain G4/5 specific (Hagg et al., 1997) **Protein, western blotting** *Mouse*: - α2 chain rabbit polyclonal Ab, C-terminus domain G4/5 specific (Tian et al., 1997) *Pig*: - α2 chain rabbit polyclonal Ab, C-terminus domain G4/5 specific (Tian et al., 1997) *Rabbit*: - α2 chain rabbit polyclonal Ab, C-terminus domain G4/5 specific (Tian et al., 1997)
			*Rat*: - α2 chain rabbit polyclonal Ab, C-terminus domain G4/5 specific (Tian et al., 1997)
Meninges	**RNA, northern hybridization** *Human*: - α2 chain, multiple nucleotide residues screened using previously reported cDNA (Vuolteenaho et al., 1994)	Not available	**Immunofluorescence** *Mouse*: - α2 chain rabbit polyclonal Ab, C-terminus domain G1–3 specific (Sasaki et al., 2002)
	**Immunofluorescence** *Mouse*: - α2 chain rabbit polyclonal Ab, C-terminus domain G1–3 specific (Sasaki et al., 2002)
Cortex (i.e., entorhinal and piriform cortices)	Not available	Not available	**Immunofluorescence** *Rabbit*: - α2 chain rabbit polyclonal Ab, C-terminus domain G4/5 specific (Hagg et al., 1997) *Rat*: - α2 chain rabbit polyclonal Ab, C-terminus domain G4/5 specific (Tian et al., 1997) - α2 chain rabbit polyclonal Ab, C-terminus domain G4/5 specific (Hagg et al., 1997) **Protein, western blotting** *Pig*: - α2 chain rabbit polyclonal Ab, C-terminus domain G4/5 specific (Tian et al., 1997)
Olfactory bulb	**RNA, northern hybridization** *Human*: - α2 chain, multiple nucleotide residues screened using previously reported cDNA (Vuolteenaho et al., 1994)	Not available	**Immunofluorescence** *Rabbit*: - α2 chain rabbit polyclonal Ab, C-terminus domain G4/5 specific (Hagg et al., 1997)
Pericytes	Not available	**Immunofluorescence** *Mouse*: - α2 chain rat mAb, N-terminus specific (Menezes et al., 2014) - α2 chain rat mAb, N-terminus specific (De La Fuente et al., 2017) **Protein, western blotting** *Rat* - α2 chain rat mAb, N-terminus specific (De La Fuente et al., 2017)	**Immunofluorescence** *Mice*: - α2 chain rat mAb, N-terminus specific (De La Fuente et al., 2017) **RNA, *in situ* hybridization** *Rat* - α2 chain rat mAb, N-terminus specific (De La Fuente et al., 2017)
Retina	**RNA, northern hybridization** *Human*: - α2 chain, multiple nucleotide residues screened using previously reported cDNA (Vuolteenaho et al., 1994)	Not available	Not available
Spinal cord	**Immunofluorescence** *Mouse*: - α2 chain rabbit polyclonal Ab, C-terminus domain G1–3 specific (Sasaki et al., 2002) *Human*: - α2 chain rabbit polyclonal Ab, C-terminus domain G4/5 specific; α2 chain mouse mAb, 80 kDa fragment specific (Liesi et al., 2001)	Not available	Not available
Thalamus and hypothalamus	Not available	Not available	**Immunofluorescence** *Rabbit*: - α2 chain rabbit polyclonal Ab, C-terminus domain G4/5 specific (Hagg et al., 1997) *Rat*: - α2 chain rabbit polyclonal Ab, C-terminus domain G4/5 specific (Hagg et al., 1997)
Tanycytes	Not available	Not available	**Immunofluorescence** *Rabbit*: - α2 chain rabbit polyclonal Ab, C-terminus domain G4/5 specific (Hagg et al., 1997) *Rat*: - α2 chain rabbit polyclonal Ab, C-terminus domain G4/5 specific (Hagg et al., 1997)
Ventricular-subventricular zone	**Immunofluorescence** *Mouse*: - α2 chain (Campos et al., 2004)	**Immunofluorescence** *Mouse*: - α2 chain (Campos et al., 2004)	Not available
	- α2 chain rat mAb, N-terminus specific (Lathia et al., 2007) *Rat*: - α2 chain (Campos et al., 2004)	- α2 chain rat mAb, N-terminus specific (Relucio et al., 2012) *Rat*: - α2 chain (Campos et al., 2004)

Note: *postnatal day 0–20. **postnatal day 21 or greater.

### Astrocytes and the Blood-Brain Barrier

Laminin-α2 is highly expressed by astrocytes (Sixt et al., 2001; Menezes et al., 2014; Yao et al., 2014; Hannocks et al., 2018) and pericytes (Yousif et al., 2013; Menezes et al., 2014; De La Fuente et al., 2017) in mouse brains. However, less is known about laminin-α2 at the blood-brain barrier (BBB) in human brains. It has been hypothesized that the absence of laminin-α2 leads to an impaired BM of the BBB, which in turn leads to a disruption of the BBB’s selective transport and filtration properties (Villanova et al., 1996, 1997). In an electron microscopy immunolabeling study examining the localization of laminin-α2 protein in five adult brains of at least 27 years old, as well as in one newborn brain (1 day old), laminin-α2 protein exclusively localized to the basal lamina of all cerebral blood vessels and was not detected in the meningeal or choroid blood vessels (Villanova et al., 1997). However other studies went on to find laminin-α2 expression in other areas of the CNS (see ahead sections; Hagg et al., 1997; Tian et al., 1997; Liesi et al., 2001; Colognato and Tzvetanova, 2011; Nascimento et al., 2018; Sato et al., 2019). The inability to detect laminin-α2 protein in other areas of the CNS may have been due to the limited capacity to detect laminins by immuno-EM from human post-mortem brain tissue. Another limitation of this study is the small sample size and the use of only one neonatal brain, as laminin expression is more abundant during the embryonic and neonatal period than in adulthood. However, given that meningeal and choroid blood vessels do not have a BBB, while cerebral blood vessels do, the expression pattern in this human study is consistent with the hypothesis that laminin-α2 is a key component at the BBB (Villanova et al., 1997).

### Hippocampal Dendritic Spines

Similar to muscle, peripheral nerve, and placental tissue, laminin-α2 protein is also found in the cortex and dendritic spines of hippocampal neurons, where it can be separated by SDS-PAGE into two fragments of 80 and 300 kDa (Tian et al., 1997). The 300 kDa fragment contains the short arm and coil-coil region of laminin-α2 protein, while the 80 kD contains a large part of the G domain, which has the interaction sites for dystroglycan and α7β1 integrin (Ehrig et al., 1990). The 300 kDa fragment only appeared in hippocampal extracts and was not detected in synaptosomes and neuronal cultures, which suggests the 300 kDA fragment could be non-neuronal or possibly that laminin-α2 was preferentially degraded in particular locations (Tian et al., 1997). Lysates from the hippocampus and cortex also revealed high levels of 160 and 140 kDa laminin-α2 protein fragments, which were at higher levels during active synaptogenesis. In the adult brain, only an 80 kDa laminin-α2 protein fragment was present. Upon a cortical lesion, which results in denervation and subsequent reinnervation, laminin-α2 immunohistochemical staining closely corresponded to the immunolabeling of the synaptic marker synaptophysin, which is loss immediately after injury and is recovered during reactive synaptogenesis (Tian et al., 1997). The dynamic expression pattern of laminin-α2 associated with synapses and dendrites suggests its likely role in synaptogenesis and/or synaptic plasticity (Anderson et al., 2005), both of which could contribute to a subset of the CNS deficits seen in MDC1A patients, such as a high incidence of epilepsy.

### Cerebellar Neurons

Laminin-α2 is spatiotemporally regulated in the cerebellum, as different laminins demonstrate distinct temporal patterns (Powell et al., 1998). Cell bodies of migrating granule cells of the cerebellum stained with laminin-111 antibodies at postnatal days 1 and 6 and less pronounced by postnatal day 12 (Powell et al., 1998). Conversely, laminin-α2 immunohistochemical staining only faintly labeled granule neurons at postnatal day 1 and then was prominently found in cell bodies of Purkinje cells at postnatal days 6 and 12 (Powell et al., 1998). Cerebellar granule cells cultured on Lm-111 and Lm-211 both stimulate neurite outgrowth, so the functional significance of differential regulation of laminin isoforms during cerebellar development remains unclear (Powell et al., 1998).

### Neurons and Glial Cells of the Spinal Cord

Immunofluorescence labeling of the BM surrounding the mouse spinal cord reveals α2-containing laminin(s) protein at 11.5 days in embryonic development (Sasaki et al., 2002). In addition, Liesi et al. (2001) used immunofluorescence, western blotting, and RT-qPCR to reveal both laminin-α2 protein and mRNA in the human fetal brain and spinal cord. While lysates from spinal cord neuronal and mixed glia cultures contained laminin-α2 protein, appearing as a 300 kDa band and a 220 kDa doublet, only mixed glial cultures contained laminin-α2 as a single 300 kDa band (Liesi et al., 2001). Interestingly, the 300 kDa α2 chain protein fragment was also previously reported as non-neuronal in hippocampal and cortical extracts (Tian et al., 1997). Why there are different laminin-α2 banding patterns by SDS-PAGE in different tissue locations remains unclear, but it has been speculated as possibly arising from either differential glycosylation, differential proteolysis, or even different splicing isoforms. Further analysis is needed to better understand the expression and processing of laminin-α2 in the brain and spinal cord.

### Laminin-α2 Expression in the Postnatal and Adult Brain

Aside from the BBB, laminin-α2 expression in the postnatal and adult brain occurs in locations associated with adult neuro- and gliogenesis, and in regions associated with ongoing progenitor cell migration, such as the olfactory bulb. In the adult CNS, neurogenesis is largely confined to the dentate gyrus of the hippocampus and the ventricular-subventricular zone of the lateral ventricles (VZ-SVZ), as well as in other ventricle-associated regions. For example, laminin-α2 immunofluorescence labeling is found in the dentate gyrus and CA3 and CA4 of the adult rat hippocampus and limbic structures (Hagg et al., 1997). In the adult rabbit brain, laminin-α2 is found in similar regions although more pronounced than in rats (Hagg et al., 1997). Laminin-α2 staining was described as noticeably labeling neuronal and dendritic processes and possibly synapses as in certain areas (i.e., thalamus and hypothalamus) the staining appeared bouton-like (Hagg et al., 1997). Consistent with this pattern, another study reported laminin-α2 protein in mouse synaptosomes from the hippocampus (Tian et al., 1997). Also, laminin-α2 protein immunofluorescence staining was reported to be prominent in tanycytes (specialized ependymal cells) of the third ventricles and ensheathing cells (specialized glia) of the olfactory bulb (Hagg et al., 1997). In the adult VZ-SVZ, laminin-α2 protein is found in fractones, which are considered an atypical BM-like ECM structure close to the ventricular surface, in close proximity to both neural stem cells (NSCs) and ependymal cells (Nascimento et al., 2018). Indeed laminin-α2 is localized to both the embryonic (Lathia et al., 2007) and early postnatal VZ-SVZ NSC niche (Relucio et al., 2012), where it has been shown to influence NSC output (Loulier et al., 2009; Relucio et al., 2012) which will be discussed in the next section.

### Laminin-α2 Expression in the Adult Brain: Changes in Response to Injury

Laminin-α2 protein is found in the adult hippocampus (Hagg et al., 1997; Tian et al., 1997), and evidence suggests that its expression is upregulated in the hippocampus after injury (Tian et al., 1997). In the developing rat hippocampus, laminin-α2 immunoreactivity increases during synaptogenesis, and upon a cortical lesion, which results in denervation and subsequent reinnervation, laminin-α2 immunoreactivity closely corresponds to the immunolabeling of the synaptic marker synaptophysin (Tian et al., 1997). These findings suggest that an α2-containing laminin has roles in synaptogenesis and synaptic plasticity, which we will discuss ahead when describing synaptic plasticity deficits in an MDC1A mouse model.

Pericytes, cells that help regulate the BBB by regulating microvascular blood flow, were found to respond to a demyelinating injury by secreting laminin-α2, which in turn promoted oligodendrocyte precursor cell (OPC) differentiation and the ability to repair myelin (De La Fuente et al., 2017). In the perinatal period OPCs are responsible for producing pre-myelinating oligodendrocytes, which later mature and become myelinating oligodendrocytes (van Tilborg et al., 2018). Since mesenchymal stem cell-conditioned medium is known as a strong inducer of OPC differentiation (Jadasz et al., 2013), and pericytes share similar features as mesenchymal stem cells (MSCs), pericyte conditioned medium was assessed and found to similarly promote OPC differentiation (De La Fuente et al., 2017). OPCs were subsequently cultured in pericyte conditioned medium pre-incubated with a laminin-α2 blocking antibody, which attenuated its effect on OPC differentiation (De La Fuente et al., 2017). Thus, a model was proposed in which pericytes produce α2-containing laminins as paracrine factors, similar to what occurs in MSCs. In a similar assessment, pericytes were found to influence the ability of NSCs to generate progenitors of an oligodendrocyte fate by producing α2-containing laminin proteins (Silva et al., 2019). Such a role for laminin-α2 in oligodendrocyte lineage development is consistent with findings from several mouse models of MDC1A demonstrating defective or delayed developmental myelination, to be discussed ahead (Chun et al., 2003; Relucio et al., 2009, 2012).

## The Role of LAMA2 in the CNS: Insights From MDC1A Mouse Models

Using a variety of MDC1A mouse models, laminins have been found to regulate NSC proliferation (impacting both neurogenesis and gliogenesis), neuronal migration, axon outgrowth, synaptogenesis, and retinal development (Figures 2, 3).

### MDC1A Mouse Model Overview

Several mouse models of MDC1A exist, with distinct Lama2 expression properties and phenotypes (Table 2; reviewed in this issue; Gawlik and Durbeej, 2020). *dy/dy* mice have a spontaneous mutation in a non-coding region of the Lama2 gene that results in substantially reduced laminin-α2 levels, causing muscular dystrophy, nervous system involvement, and premature death (Michelson et al., 1955). *dy*^2J^/*dy*^2J^ mice have a point mutation that causes a splicing change, resulting in a truncated laminin-α2 chain that lacks the N-terminal LN domain (Xu et al., 1994; Sunada et al., 1995), a domain that is critical for mediating laminin-laminin polymer interactions (Colognato and Yurchenco, 1999; Yurchenco et al., 2018). Similar to *dy/dy* mice, *dy*^2J^/*dy*^2J^ mice have muscular dystrophy and a shortened lifespan, although *dy*^2J^/*dy*^2J^ mice are healthier and live longer than do *dy/dy* mice. The *dy*^w^/*dy*^w^ mouse was generated by homologous recombination in embryonic stem cells and was initially thought to be a full Lama2 knock-out mouse, but later found to express low levels of laminin-α2. The *dy^w^/dy^w^* mouse exhibits severe muscular dystrophy and survival is reduced to a range of 5–16 weeks (Willmann et al., 2017). Lastly, *dy*^3k^/*dy*^3k^ mice are Lama2 knockout mice, having a complete loss of laminin-α2 expression, which leads to severe muscular dystrophy and reduced life expectancy (~5 weeks; Miyagoe et al., 1997).

**Table 2:** LAMA2 mouse models.

Lama2 Mouse	Mutation	Laminin-α2 levels	CNS Involvement		
			Myelination deficits	BBB Deficits	Other
***dy/dy*** (Michelson et al., 1955)	Spontaneous mutation	Substantially reduced	Impaired oligodendrogenesis and myelination (Chun et al., 2003; Relucio et al., 2009).	Unknown	Elevated audiometric threshold response, degeneration of cochlear and vestibular structures (Pillers et al., 2002). Atrophy of motor neurons and abnormal neurotrophic factor expression in CNS (Sakuma et al., 2002)
***dy*^2J^/*dy*^2J^** (Meier and Southard, 1970)	Spontaneous mutation; abnormal splicing and subsequent instability leads to a truncated protein that lacks the N-terminus	Expressed but lacking LN domain; modest reduction in levels	Unknown	Unknown	Long term plasticity was disrupted (Anderson et al., 2005).
***dy*^W^/*dy*^W^** (Kuang et al., 1998)	Targeted knock-out; truncated protein	Very low to absent	Unknown	Unknown	
***dy*^3k^/*dy*^3k^** (Miyagoe et al., 1997)	Targeted knock-out	Absent	Impaired oligodendrogenesis and myelination (Relucio et al., 2012).	BBB dysfunction and increased permeability (Menezes et al., 2014).	Impaired NSC proliferation and attachment within the ventricular zone (Loulier et al., 2009; Relucio et al., 2012).

*dy/dy* mice exhibit myelination defects of both the peripheral nervous system (PNS; Harris et al., 1972) and CNS (Chun et al., 2003; Relucio et al., 2009), impaired sodium channel clustering at NMJs (Occhi et al., 2005), and aberrant neural stem cell function (Loulier et al., 2009). Similar to dy/dy mice, *dy*^3k^/*dy*^3k^ mice have impaired oligodendrogenesis (Relucio et al., 2012). *dy*^3k^/*dy*^3k^ mice also have BBB dysfunction and increased permeability (Menezes et al., 2014). Lastly, there is currently no information regarding the CNS in *dy*^w^/*dy*^w^ mice. The CNS findings from the *dy/dy*, *dy*^2J^/*dy*^2J^, and *dy*^3k^/*dy*^3k^ mice will be expanded upon in the following section.

### Laminin-α2 Regulates Synaptic Plasticity

Despite the occurrence of epilepsy in many MDC1A patients, there is currently limited knowledge regarding the role of laminin-α2 in CNS synapse function. However, *dy^2J^/dy^2J^* mice were found to have disruptions in long term neuronal plasticity, despite having no change in basal synaptic transmission and paired-pulse stimulation (Anderson et al., 2005). Using cerebellar slice preparation to invoke long term depression, over half of the cells examined in *dy*^2J^/*dy*^2J^ mice displayed long term depression that was significantly reduced compared to that in control mice. Furthermore, nearly 1/3 of the sampled cells exhibited aberrant long-term potentiation, which was not seen in controls. Since there was not a difference in pre-synaptic mediated short-term plasticity, this study proposed that the differences in long-term plasticity originate post-synaptically.

There is additional support for the DGC being involved in synaptic plasticity. It is already known that the DGC is necessary for stabilizing the clustering of acetylcholine receptors at the NMJ (Jacobson et al., 2001; Nishimune et al., 2008). In the hippocampus, dystroglycan co-localizes with almost all GABA_A_ receptor α1 clusters (Pribiag et al., 2014). Disruption of the DGC by either loss of dystrophin, which anchors the complex to the actin cytoskeleton, or mutations that result in hypoglycosylation of α-dystroglycan, result in fewer dystroglycan and α1 clusters in the hippocampus (Kueh et al., 2011; Pribiag et al., 2014). Furthermore, dystroglycan is required to recruit additional GABA_A_ receptors at the postsynaptic site during homeostatic scaling up (Pribiag et al., 2014). Homeostatic scaling up of inhibitory synaptic strength is a critical physiological mechanism to maintain balance between excitation and inhibition of neuronal activity, and the loss of this response by GABA_A_ receptors contributes to the development of epilepsy (Chuang and Reddy, 2018). Lastly, the loss of inhibitory synaptic protein 1 (InSyn1), which binds to both the DGC and to gephyrin, a GABA receptor anchoring protein, also leads to poor dystroglycan and GABA_A_ receptor α1 clustering at the postsynaptic site in the hippocampus (Uezu et al., 2019).

Together these findings support the hypothesis that laminin and the DGC impact inhibitory synapses and that mutations affecting either can alter neuronal activity.

### Laminin-α2 Regulates the Attachment and Proliferation of Neural Stem Cells

Laminins are expressed in the developing (Campos et al., 2004; Lathia et al., 2007), postnatal (Relucio et al., 2012; Nascimento et al., 2018) and adult ventricular-subventricular zone (VZ-SVZ; Shen et al., 2008; Tavazoie et al., 2008; Nascimento et al., 2018; Sato et al., 2019). In embryonic development, NSCs of the VZ-SVZ express laminin-α2 receptors such as α6β1 integrin and dystroglycan during a critical time when NSCs undergo expansion via asymmetrical division for cortical neuronal development (Lathia et al., 2007). During this time, laminin-α2 protein is also present in the VZ-SVZ (Lathia et al., 2007). *dy^3k^/dy^3k^* mice show impaired NSC proliferation, cellular position, and attachment within the VZ-SVZ, a phenotype that is similar to what occurs when β1 integrin functions are blocked by antibodies injected into the lateral ventricle (Loulier et al., 2009). Laminin-α2 also has a role in the postnatal VZ-SVZ. In both laminin α2-deficient *dy/dy* mice and laminin α2-absent *dy*^3k^/*dy*^3k^ mice, defects in oligodendrocyte production and maturation were observed (Chun et al., 2003; Relucio et al., 2009, 2012). In the case of *dy*^3k^/*dy*^3k^ mice, increased death of glial progenitors was observed in the neonatal VZ-SVZ, suggesting a lack of appropriate survival cues (Relucio et al., 2012). Laminin-α2 also has a role in the midbrain dopaminergic neuron progenitor niche (Ahmed et al., 2019). In the ventral midbrain, laminin-α2 interactions with integrins regulate the proliferation and survival of progenitors (Ahmed et al., 2019). In the *dy*^3k^/*dy*^3k^ mouse the absence of laminin-α2 results in increased apoptosis and depletion of the progenitor pool, leading to a reduction in later-born ventral tegmental area (VTA) neurons (Ahmed et al., 2019). In this study (Ahmed et al., 2019) it was hypothesized that the loss of VTA neurons, which normally innervate the hippocampus and prefrontal cortex (Morales and Margolis, 2017), provides a mechanism for the hypoplasia of the brainstem seen in some MDC1A patients. They also propose (Ahmed et al., 2019) that loss of laminin-mediated interactions in this neurogenic niche could contribute to the autism-like behaviors seen in some related muscular dystrophies such as DMD (Ricotti et al., 2016), although to date there are no reports of increased incidence of autism-like behaviors in MCD1A itself.

### Laminin-α2 Regulates Neurite Outgrowth and Axonal Pathfinding

The detection of laminin-α2 in the developing visual pathways is spatiotemporal, occurring as retinal ganglion cell (RGC) growth cones extend their projection into the brain (Cohen and Johnson, 1991; Morissette and Carbonetto, 1995). In vitro, embryonic RGC neurite outgrowth is mediated by α6β1 integrin receptors and α2-containing laminin (Cohen and Johnson, 1991). Both RGCs and astrocytes from developing optic nerves are capable of synthesizing α2-containing laminin, although thus far this capability has only been directly demonstrated in culture (Morissette and Carbonetto, 1995). After the development of the retina and optic nerve, laminin-α2 protein levels are reduced but still detectable by immunofluorescence in the adult mouse (Morissette and Carbonetto, 1995).

Primary oligodendrocyte cultures from mice demonstrated a similar neurite outgrowth-like response to Lm-211(Buttery and ffrench-Constant, 1999; O’Meara et al., 2013; Michalski et al., 2016). Depletion of integrin-linked kinase (ILK), an adapter protein that interacts with β1-integrin to regulate cytoskeletal dynamics in growth cone extension, results in impaired oligodendrocyte process extension and ability to form myelin membrane upon axonal contact (O’Meara et al., 2013). When cultured oligodendrocytes from ILK^−/−^ mice are grown on Lm-211, ILK^−/−^ oligodendrocytes have less branching and severely stunted branches when compared to control oligodendrocytes, which are highly branched (Michalski et al., 2016). These results suggested that coordination between the ECM (i.e., Lm-211 binding) and oligodendrocyte cytoskeleton dynamics (i.e., β1 integrin and ILK) is necessary for oligodendrocyte process extension. Oligodendrocytes that lack expression of the Lm-211 ligand, dystroglycan, also have deficits in oligodendrocyte branching (Eyermann et al., 2012), suggesting either coordination or redundancy in the receptors required for the ability of Lm-211 to regulate process dynamics in the developing CNS.

### Laminin-α2 Regulates the Integrity of the Blood-Brain Barrier

Laminin-α2 is found in BMs of the blood-brain barrier, or BBB (Villanova et al., 1997), whose correct function requires contributions from cerebral blood vessels, astrocytes, and pericytes. In the absence of laminin-α2, there is increased permeability of the BBB dysfunction as well as several cellular and molecular changes associated with BBB dysfunction (Menezes et al., 2014). For instance, *dy^3k^/dy^3k^* mice have reactive astrogliosis, altered gliovascular morphology, and decreased pericyte coverage along the cerebral vasculature throughout postnatal development (Menezes et al., 2014). The Lm-211 receptor, dystroglycan, is expressed in BBB astrocytes where it anchors aquaporin channels (AQP4) at astrocytic endfeet; this localization is crucial to water homeostasis at the BBB (Lien et al., 2012). *dy*^3k^/*dy*^3k^ cerebral cortices have decreased AQP4 immunoreactivity along astrocytic endfeet, although overall AQP4 levels were not significantly affected (Menezes et al., 2014), suggesting that in the absence of α2-containing laminins, AQP4 fails to appropriate localize. Consistent with BBB findings in *dy*^3k^/*dy*^3k^ mice, the loss of laminin γ1 chain in pericytes, which will indirectly prevent the expression of Lm-211, results in a similar phenotype with BBB disturbances (Gautam et al., 2016).

### Loss or Deficiency of Laminin-α2 Impairs Oligodendrogenesis and Myelination

Laminin-α2 protein is found in the postnatal ventricular/subventricular zone (VZ-SVZ; Campos et al., 2004), the largest neuro/gliogenic niche of the postnatal brain. In the complete absence of laminin-α2 (*dy^3k^/dy^3k^* mice), there is a reduction in the thickness of the dorsal VZ-SVZ, suggesting either decreased cell division, increased cell death, or a combination thereof (Relucio et al., 2012). In both the embryonic (Loulier et al., 2009) and postnatal (Relucio et al., 2012) VZ-SVZ, *dy*^3k^/*dy*^3k^ mice have impaired NSC arrangements, presumably due to failed or altered cellular attachments. In the VZ-SVZ at postnatal day 1, NSC densities are unchanged but there are fewer OPCs in *dy*^3k^/*dy*^3k^ mice compared to control littermates, accompanied by increased OPC death (Relucio et al., 2012). This deficit seems to be time-sensitive, as at postnatal day 8 *dy*^3k^/*dy*^3k^ mice rebound and have *more* OPCs than control mice, along with a return to normal levels of cell death (Relucio et al., 2012). Beyond the NSC niche of the VZ-SVZ, laminin-α2 also regulates the development of neuronal and glial progenitor cells. Similar to how laminin-α2 promotes the survival of dopaminergic progenitors (Ahmed et al., 2019), laminin-α2 regulates the number of OPCs in the developing corpus callosum (Relucio et al., 2012), the nearest white matter tract to the dorsolateral VZ-SVZ germinal niche. Laminin-α2 also promotes OPC differentiation in the corpus callosum and other white matter regions. For example, despite increased OPC densities during early cortical myelination in *dy*^3k^/*dy*^3k^, these mice have significantly fewer mature oligodendrocytes as well as delayed myelination (see ahead). Together these data suggest that α2-containing laminins are important during a critical stage of oligodendrocyte development.

Laminin-α2 protein immunoreactivity is transiently found in axon tracts undergoing myelination (Milner and Ffrench-Constant, 1994; Colognato et al., 2002) and myelinating oligodendrocytes express the laminin-α2 receptor integrin α6β1 (Milner and Ffrench-Constant, 1994; Colognato et al., 2002). In the *dy/dy* mouse (sharply reduced laminin-α2 levels) there is a reduction in both mature oligodendrocytes and myelin content in the corpus callosum, a major white matter tract. Ultrastructural analysis using electron microscopy, the gold standard for examining myelin structure, revealed an increased g-ratio (i.e., thinner myelin) in the axons in the corpus callosum, optic nerve, brainstem and the cerebellum, but not from the spinal cord (Chun et al., 2003; Relucio et al., 2009). In addition to thinner myelin, *dy/dy* mice have other indications of impaired myelination such as regions of noncompacted myelin (Chun et al., 2003; Relucio et al., 2009). This suggests that the loss of laminin-α2 either diminishes the capacity of OPCs to differentiate into mature myelinating oligodendrocytes or alternatively, OPCs differentiate but oligodendrocytes are unable to proceed with normal myelination, i.e., have impaired myelination capacity. Given that *dy*^3k^/*dy*^3k^ mice have fewer mature oligodendrocytes the former is a likely component but the latter has not been ruled out as a contributing factor. In agreement with findings in *dy*^3k^/*dy*^3k^, *dy/dy* mice have an increase in OPC markers and a decrease in mature oligodendrocyte markers, determined by both immunohistochemical staining and western blotting (Relucio et al., 2009).

While it not entirely clear how α2-containing laminins regulate oligodendrocyte development, some mechanistic details have emerged. Disturbances in the regulation of Fyn, a Src family kinase required for myelination, were reported in *dy/dy* mice (Relucio et al., 2009). The level of phosphorylated Fyn at its Y529 site is significantly increased in the cerebral cortex of *dy/dy* mice, as are levels of Csk, a negative regulator of Fyn that phosphorylates Fyn at the Y529 position to render it inactive. Thus, it appears that disturbances in Fyn activity are a strong contender to contribute to delayed oligodendrocyte maturation and myelination in laminin-α2 deficiencies (Relucio et al., 2009). This hypothesis is supported by work using cultured wildtype OPCs, in which α2-containing laminins accelerate OPC development into oligodendrocytes (Buttery and ffrench-Constant, 1999), an effect that was later shown to be blocked by PP2, a Src kinase inhibitor (Relucio et al., 2009). In addition, changes in oligodendrocyte numbers are not likely due to increased oligodendrocyte death since no differences in oligodendrocyte death were observed in *dy/dy* mice (Relucio et al., 2009). In agreement with this observation, while increased death of OPCs occurred in germinal zones, no change in oligodendrocyte death was observed in *dy*^3k^/*dy*^3k^ mice (Relucio et al., 2012).

Lastly, although peripheral myelination is not the primarily focus of this review, it is important to note that Lm-211 is also a major component of the BM in the PNS. Schwann cells, the myelinating cells of the PNS, express laminin receptors such as dystroglycan and integrins, which interact with Lm-211 to anchor the outer myelin membrane to the ECM and also activate pathways involved in Schwann cell maturation and myelination of peripheral nerves (Nakagawa et al., 2001; Patton et al., 2008; Court et al., 2009; Homma et al., 2011; Heller et al., 2014; Petersen et al., 2015; Ghidinelli et al., 2017). Defects associated with loss of LAMA2 expression in the PNS are detailed further in a review in this issue (Previtali and Andrea Zambon, 2020).

## MDC1A: Lessons From Related Developmental Disorders

Collectively, muscular dystrophies are characterized by dystrophic muscle fibers: hypercontracted and degenerating fibers, increased fiber size variability, and increased connective tissue infiltration. While CMDs present in the first months to years of life, other muscular dystrophies such as limb-girdle muscular dystrophy have later onsets. CMDs can furthermore present with neurological symptoms such as ventricular enlargement, abnormalities in brain morphology and white matter changes, which can also be highly heterogeneous. It should be noted that non-MDC1A CMD patients may also have reduced laminin-α2 levels, as a reduction in laminin-α2 protein levels may be from primary deficiency, i.e., MDC1A, or from secondary reduction caused by mutations not in the LAMA2 gene. Secondary reductions are thought to reflect the interplay between α2-containing laminin proteins and members of the DGC, where the loss of one binding partner in the DGC has been observed to cause changes in the localization or levels of other complex members (Muntoni et al., 1998; Brockington et al., 2001). Ahead we will discuss key features of other neurodevelopmental disorders that share similar CNS clinical features as MDC1A, and how these similar features could point to shared cellular and molecular mechanisms.

### Selected Dystroglycanopathies

**Figure 4:**
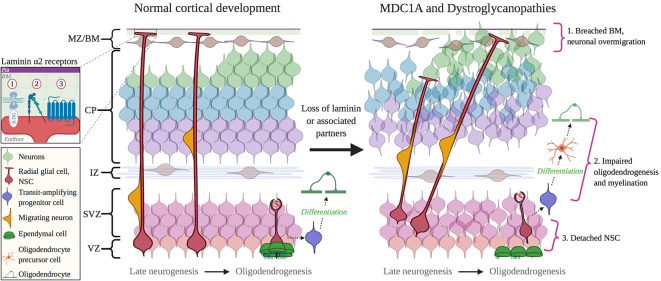
Cortical dysplasia and NSC dysfunction in Congenital Muscular Dystrophies (CMD). *Left*: normal cortical development. *Right*: abnormal cortical development in CMD. 1. Breached BM caused by mutations in DAG1 and GPR56 genes, or abnormal glycosylation by fukutin, FKRP, and LARGE1. 2. Impaired oligodendrogenesis due to loss of LAMA2 and DAG1 expression or function. 3. The detachment of NSCs due to the loss of a functional interaction between laminin α2 and β1 integrins. Inset: receptors mediate interactions between radial glial cell endfeet and with laminin α2: 1, Dystroglycan; 2, Integrin; 3, GPR56 and (possibly) GPR126. Abbreviations: BM, basement membrane; CP, cortical plate; IZ, intermediate zone; MZ/BM, Marginal Zone/Basement Membrane; NSC, neural stem cell; SVZ, subventricular zone; VZ, ventricular zone.

Neuronal migration defects are seen in several CMDs that result from mutations impairing dystroglycan function (Figure 4). Dystroglycan mRNA is found in selected radial glial cells in the VZ-SVZ, while dystroglycan protein is mostly localized in the radial glial endfeet, where it mediates radial glial anchoring to the pial BM (Myshrall et al., 2012). Loss of dystroglycan function in radial glia results in the over-migration of neurons due to disruptions in the pial BM, a phenotype known as cobblestone lissencephaly (Myshrall et al., 2012). Primary dystroglycanopathies resulting from mutations in the DAG1 gene that encodes dystroglycan are quite rare, however secondary dystroglycanopathies, of which there are multiple types, occur due to mutations in multiple genes involved in the glycosylation of α-dystroglycan (selected examples discussed ahead).

Fukuyama congenital muscular dystrophy (FCMD) is caused by mutations in the FKTN gene, which encodes for fukutin. Closely related is Congenital Muscular Dystrophy Type 1C (MDC1C), resulting from mutations in FKRP, which encodes the fukutin-related protein. Both Fukutin and FKRP are Rbo5P transferases responsible for the post-translational modification of α-dystroglycan (Kanagawa et al., 2016). Impaired glycosylation of α-dystroglycan prevents its binding to extracellular ligands including Lm-211 but also to other ECM proteins that contain LG domains (e.g., perlecan, neurexin; Kanagawa et al., 2016). Similar to MDC1A, both FCMD and MDC1C are multisystem disorders that present with delays in neurodevelopment, cortical dysgenesis, and ocular abnormalities (e.g., myopia and retinal degeneration; Tsutsumi et al., 1989; Angelini, 2016).

The LARGE1 gene also encodes for a glycosyltransferase that is critical for the glycosylation of α-dystroglycan (Jimenez-Mallebrera et al., 2005). Mutations in LARGE1 result in the hypoglycosylation of α-dystroglycan, subsequently impairing α-dystroglycan binding to Lm-211 (Longman et al., 2003). Mutations in LARGE1 cause Congenital Muscular Dystrophy Type 1D (MDC1D), which can present with cognitive deficits that are accompanied by structural brain abnormalities including abnormal white matter and neuronal migration defects that manifest as “cobblestone” ectopias (Longman et al., 2003; Montanaro and Carbonetto, 2003). Mutations in LARGE1 can also result in WWS, which can also be caused by mutations in FKRP and, most notably, POMT1. In addition to being a more severe muscular dystrophy, WWS is characterized by cobblestone lissencephaly and ocular malformations, which include optic nerve hypoplasia and retinal malformation (Dobyns et al., 1989). A final example is Muscle Eye Brain disease (MEB), caused by a mutation in the POMGnT1 gene, which is also responsible for correct glycosylation of α-dystroglycan (Yis et al., 2014). MEB presents with similar symptoms as other CMDs (i.e., hypotonia and muscle weakness), but unlike classical MCD1A, MEB presents with severe ophthalmological findings such as severe myopia (>10 diopters), glaucoma, retinal malformation and uncontrolled eye movements (Santavuori et al., 1989; Haltia et al., 1997; Yis et al., 2014). Similar to MDC1A, however, MEB is usually accompanied by epilepsy (Santavuori et al., 1989).

Not surprisingly, given the central role of the laminin-DGC connection in skeletal muscle, many dystroglycanopathies share features with MDC1A. The neurological features of these diseases also have many similarities with MDC1A. Despite this, there are differences, particularly in the degree of severity in both neurological problems and CNS structural deficits. The basis of these differences remains unknown, but the fact that α2-containing laminins have other roles besides interacting with dystroglycan, and dystroglycan has multiple LG-containing ECM protein partners in the brain (e.g., neurexin), likely contributes. Overall, the precise network and interplay of laminin- and dystroglycan-interactions in the brain is incomplete and will require a concerted effort to understand, for example, efforts to interrupt the expression of both proteins in a temporal and cell-specific fashion during neurodevelopment, as well as the to express LAMA2 with domain-specific mutations designed to abolish particular binding interactions.

### GPR126-Related Cortical Dysplasia

An additional receptor for α2-containing laminins is the adhesion G protein-coupled receptor, GPR126. Upon ECM binding, adhesion G protein-coupled receptors (GPCRs) undergo autoproteolysis that results in two cleaved products: an N-terminal fragment, which contains the GAIN domain responsible for autoproteolysis, and a seven-transmembrane-containing C-terminal fragment (Langenhan et al., 2013). GPR126 is required in Schwann cells, the myelinating cells of the PNS, where it acts by increasing cAMP levels to activate protein kinase A, which initiates the upregulation of transcription factors required for Schwann cell myelination (Glenn and Talbot, 2013). Lm-211 interacts with the GAIN domain in the N-terminal GPR126 fragment to either promote myelination or suppress myelination, as well as favor radial sorting of axons in the PNS (Petersen et al., 2015). Although it remains unknown if GPR126 interacts with α2-containing laminins in the CNS, the clinical phenotype of patients with mutations in GPR126 indicates that GPR126 likely interacts with ECM proteins in the brain. For example, a recent study described two patients with GPR126 mutations with intellectual disabilities (Hosseini et al., 2019). The first patient had normal motor development until 13 months of age when they developed a generalized seizure. At 16 years old, the patient had a low IQ estimated between 20–25, and MRI revealed cerebellar hypoplasia (Hosseini et al., 2019). The second patient developed refractory epilepsy by 12 months of age and again had a low IQ, estimated between 20–25 (Hosseini et al., 2019). Both patients had mutations in the seven-transmembrane-containing C-terminal fragment, which is responsible for increasing cAMP to induce Schwann cell myelination (Langenhan et al., 2013), but interestingly, these patients had CNS symptoms, rather than peripheral neuropathy. Given the overlap in neurological deficits between MDC1A patients and patients with GPR126 mutations, it seems likely that failed interactions between GPR126 and α2-containing laminins underlie at least some of the CNS features of patients with GPR126 mutations.

### GPR56- Related Cortical Dysplasia

Mutations in GPR56, another adhesion GPCR for α2-containing laminins, also cause neurological symptoms. Like all GPCRs, GPR56 contains a seven-transmembrane-containing domain and has a long N-terminus extension similar to GPR126. Interestingly, GPR56 contains a mucin-rich domain similar to that found in dystroglycan (Piao et al., 2004). *In situ* hybridization experiments in mice demonstrated that GPR56 localizes to the VZ-SVZ during embryonic development but minimally in other areas of the cortex, which suggests GPR56 involvement in neurogenesis (Piao et al., 2004). Mutations in the gene encoding GPR56 result in bilateral frontoparietal polymicrogyria, which is characterized by abnormal cortical lamination and gyral organization (Piao et al., 2004; Bahi-Buisson et al., 2010). GPR56 is localized in radial glial endfeet, and in GPR56^−/−^ mice there is rupture of the pial BM, which results in neuronal over migration (Li et al., 2008). Desai and Udani (2015) reported four cases of patients with a mutation in GPR56 with diffuse bilateral polymicrogyria in the frontoparietal lobes as well as ocular findings such as strabismus. MRI furthermore revealed diffuse white matter abnormalities in two out of four patients and frontal periventricular white matter changes in the other two patients. In a previous study of 30 patients with bilateral frontoparietal polymicrogyria, 14 were found to have mutations in GPR56 (Bahi-Buisson et al., 2010). All 14 patients had a severe cognitive delay, 7 out of 14 had eye movement abnormalities, and 12 out of 14 had epilepsy (Bahi-Buisson et al., 2010). Given that MDC1A neurological symptoms overlap with those in patients with GPR56 mutations, combined with the knowledge that GPR56 and α2-containing laminins both are required for the correct regulation of NSCs during brain development in mice, it appears very likely that GPR56 and α2-containing laminins interact during human CNS neurodevelopment.

## Conclusion

While the degree of brain involvement is highly variable in different MDC1A patients, an emerging theme is that the severity of dystrophic symptoms and neurological symptoms can be uncoupled and do not always follow a strict pattern depending on LAMA2 mutation type. However complete loss of LAMA2 expression is correlated with more severe CNS involvement. Together these themes suggest a large degree of variability in how different patterns of gene expression and function, both from patient to patient and tissue to tissue, can alter the trajectory of this complex disease. Lastly, it is increasingly clear that α2-containing laminins have diverse roles in the developing brain, both during embryonic development and during postnatal development, from ensuring correct BBB function, to the organization of the developing cortical plate, to the proper development of oligodendroglia. The precise receptor interactions as well as the precise chemical and mechanical signaling properties of α2-containing laminins in the brain are yet to be discovered but will be critical in the development and feasible timing of future avenues of intervention in neurological aspects of MDC1A as well as other CMDs with brain involvement.

## Author Contributions

AJA and HC wrote the manuscript. AJA created the figures.

## Conflict of Interest

The authors declare that the research was conducted in the absence of any commercial or financial relationships that could be construed as a potential conflict of interest.
